# Performance of artificial intelligence in diabetic retinopathy screening: a systematic review and meta-analysis of prospective studies

**DOI:** 10.3389/fendo.2023.1197783

**Published:** 2023-06-13

**Authors:** Zhibin Wang, Zhaojin Li, Kunyue Li, Siyuan Mu, Xiaorui Zhou, Yu Di

**Affiliations:** Department of Ophthalmology, Shengjing Hospital of China Medical University, Shenyang, China

**Keywords:** artificial intelligence, diabetic retinopathy, meta-analysis, diagnostic accuracy, prospective study

## Abstract

**Aims:**

To systematically evaluate the diagnostic value of an artificial intelligence (AI) algorithm model for various types of diabetic retinopathy (DR) in prospective studies over the previous five years, and to explore the factors affecting its diagnostic effectiveness.

**Materials and methods:**

A search was conducted in Cochrane Library, Embase, Web of Science, PubMed, and IEEE databases to collect prospective studies on AI models for the diagnosis of DR from January 2017 to December 2022. We used QUADAS-2 to evaluate the risk of bias in the included studies. Meta-analysis was performed using MetaDiSc and STATA 14.0 software to calculate the combined sensitivity, specificity, positive likelihood ratio, and negative likelihood ratio of various types of DR. Diagnostic odds ratios, summary receiver operating characteristic (SROC) plots, coupled forest plots, and subgroup analysis were performed according to the DR categories, patient source, region of study, and quality of literature, image, and algorithm.

**Results:**

Finally, 21 studies were included. Meta-analysis showed that the pooled sensitivity, specificity, pooled positive likelihood ratio, pooled negative likelihood ratio, area under the curve, Cochrane Q index, and pooled diagnostic odds ratio of AI model for the diagnosis of DR were 0.880 (0.875-0.884), 0.912 (0.99-0.913), 13.021 (10.738-15.789), 0.083 (0.061-0.112), 0.9798, 0.9388, and 206.80 (124.82-342.63), respectively. The DR categories, patient source, region of study, sample size, quality of literature, image, and algorithm may affect the diagnostic efficiency of AI for DR.

**Conclusion:**

AI model has a clear diagnostic value for DR, but it is influenced by many factors that deserve further study.

**Systematic review registration:**

https://www.crd.york.ac.uk/prospero/, identifier CRD42023389687.

## 1 Introduction

Diabetic retinopathy (DR) is one of the leading causes of blindness among middle-aged and older people worldwide (1, 2). According to international standards, DR is mainly divided into nonproliferative diabetic retinopathy (NPDR) and proliferative diabetic retinopathy (PDR) based on the condition of the fundus. As a common complication of diabetes, it is estimated that up to 30% of diabetic patients will eventually develop various types of DR (3), and 10% of diabetic patients are at risk of blindness (4).

Fundus color photography plays a key role in the screening of DR, which has traditionally relied on the clinical experience of ophthalmologists or retinal specialists who comprehensively evaluate the patient’s condition through routine ophthalmology examination, fundus scope, optical coherence tomography (OCT), and other methods. However, as DR is an insidious disease, most patients are not consciously aware of the existence of the disease, especially those living in areas with underdeveloped medical facilities, making it challenging for ophthalmologists to make an accurate and timely diagnosis of the patient’s condition from fundus color photography. In addition, ophthalmologists often adopt different intervention methods for DR patients with different disease states. Therefore, in order to delay the occurrence and development of DR, reduce the blinding rate, and improve the quality of life of patients, it is necessary to accurately distinguish the various types of DR at an early stage of the disease.

As deep learning technology has advanced in recent years, the application of artificial intelligence (AI) in the medical field is receiving increasing attention. It involves the analysis and evaluation of image-related data through the establishment of relevant databases and application models, and information processing (5). In the field of ophthalmology, AI is widely used in DR, age-related macular degeneration (AMD), glaucoma, cataract, and other diseases (6), and fully autonomous diagnostic systems have already been developed (7). As a potential method to assist clinical ophthalmologists in the diagnosis and treatment of DR, one of the obvious advantages of AI is its high diagnostic accuracy. Studies have shown that the accuracy of DR diagnosis can reach up to 90% by using a deep learning mode (8–10), and 80% or above with a machine learning model (11–13). Therefore, AI can effectively relieve the pressure on ophthalmologists by conducting mass image screening and improve their efficiency of ophthalmologists in the diagnosis and treatment of related diseases and complications, thus solving the problem of insufficient medical resources and promoting the comprehensive development of blindness prevention and treatment strategies. At present, diagnostic meta-analyses on the accuracy of DR detection by AI have mainly focused on a specific algorithm (14, 15). Additionally, most studies are based on the mining of publicly available datasets, which lack verification in the real world. Although these datasets are not updated in time, they are repeatedly cited in many meta-analyses. Moreover, in previous meta-analyses, most of the included studies were retrospective studies, which may cause bias in the real world setting. The present meta-analysis will be systematically investigated the performance and application status of AI in diagnosing DR based on fundus color photographs in the real world in the last five years. Furthermore, the factors that might affect the diagnostic effect of AI through subgroup analysis will be explored. Our results can further validate the role of AI in clinical decision making.

## 2 Materials and methods

### 2.1 Search strategy

Two reviewers (KL and SM) searched relevant prospective studies in the Cochrane Library, PubMed, Embase, Web of Science, and The Institute of Electrical and Electronics Engineers (IEEE) databases over the last 5 years. Each of the other reviewers re-evaluated whether the search strategy was appropriate and whether the included literature was consistent with the research purpose. The terms of our search were as follows: (“Diabetic Retinopathy” OR “Diabetic Retinopathies” OR “Retinopathies, Diabetic” OR “Retinopathy, Diabetic”, then combined these items using AND with “Artificial intelligence” OR “machine learning” OR “deep learning” OR “neural network”) AND (“diagnosis” OR “screen” OR “classification” OR “discriminate”) AND (“performance” OR “sensitivity” OR “specificity” OR “accuracy” OR “area under the curve” OR “auc”). The meta-analysis was conducted following the PRISMA (16) (**Supplementary Table S4**).

### 2.2 Study selection and eligibility criteria

Criteria for inclusion in the meta-analysis were: (1) the study was a diagnostic study; (2) the subjects were type 2 or type 1 diabetic patients with DR; (3) the diagnostic measure was AI technology, and DR was diagnosed from fundus color images; (4) the study was complete with available data on sensitivity (SE), specificity (SP), number of patients and controls, and other outcome indicators; (5) prospective research in the last 5 years. Exclusion criteria: (1) Diagnostic measures do not belong to AI technology and no AI-related algorithm was used; (2) studies with duplicate data and incomplete original data; (3) studies with incomplete or inaccessible outcome indicators. ZW and ZL selected the studies independently according to the inclusion and exclusion criteria above. If there was a difference of opinion among the reviewers, a joint consultation was held with a third reviewer (XZ) before making a decision. Here are populations, interventions, comparators, outcomes, and study designs (PICOS) in our study. Population of our research comes from type 2 or type 1 diabetic patients with DR; interventions: patients with DR are diagnosed by AI technology.; comparators: patients with DR are diagnosed by clinical doctors;.study design: the study was a diagnostic study.

### 2.3 Data extraction

After obtaining the full articles, two reviewers (ZW and ZL) independently summarized the features of the included studies and extracted outcome indicators related to the diagnostic efficacy of AI from each study. We addressed divergence between the two reviewers’ data extraction by discussion and consultation with a third investigator (KL). Reviewers directly extracted SE, SP, and number of DR patients and total participants from the included studies. These indicators were used to calculate the outcome variables for the diagnostic meta-analysis, namely true-positives (TP), false-positives (FP), false-negatives (FN), and true-negatives (TN), which were then entered into contingency tables, followed by subsequent meta-analysis. If a study contained different types of DR or different algorithms, and there were multiple contingency tables, we assumed that they were independent of each other.

### 2.4 Quality assessment

To assess the quality of the included Studies, two investigators (SM and XZ) used Quality Assessment of the Diagnostic Accuracy Studies 2 (QUADAS-2) (17) and RevMan 5.3. QUADAS-2 scale includes four bias risk assessment parts, namely patient selection, index test, reference standard, and flow and timing. Each part has two or three questions. If all the answers were “Yes”, that part was considered as low risk. Additionally, patient selection, index test, and reference standard were also evaluated in terms of the clinical applicability. If the answers to these assessment parts were “low risk”, it indicates that the included studies are less biased.

### 2.5 Data synthesis and analysis

We used MetaDiSc software (version 1.4) for the outcome variables (TP, FP, FN, TN). Summary receiver operating characteristic (SROC) plots and coupled forest plots were used to visualize the merger results. The I^2^ test and Cochrane-Q test were used to evaluate heterogeneity caused by possible non-threshold effects in this meta-analysis. If I^2^>50%, it was considered as significant heterogeneity. Subsequently, bivariate random effects model was used to calculate the pooled sensitivity, specificity, area under the curve (AUC), diagnostic odds ratio (DOR), positive and negative likelihood ratios (LR+ and LR-, respectively), among which area under the SROC curve indicates the diagnostic value of AI for DR. In order to explore how categories of DR, source of patients, sample size, country, quality of included studies and images, and different algorithms can influence the merged results, we performed subgroup analysis according to the above factors.

We used the midas package in STATA14.0 to conduct a sensitivity analysis of the included studies to explore the source of heterogeneity. Furthermore, the incidence rate of DR (30%) was taken as the prior probability, and the posterior probability was calculated according to the summarized LR+ and LR-. The results were visualized in STATA14.0 and displayed with fagan plots. We have assess publication bias by plotting Deek’s funnel plot. The funnel plot is asymmetric when significant publication bias is present. All statistical results were considered significant if the two-tailed p value<0.05.

## 3 Results

### 3.1 Selection and characteristics of the eligible studies

**Figure 1:**
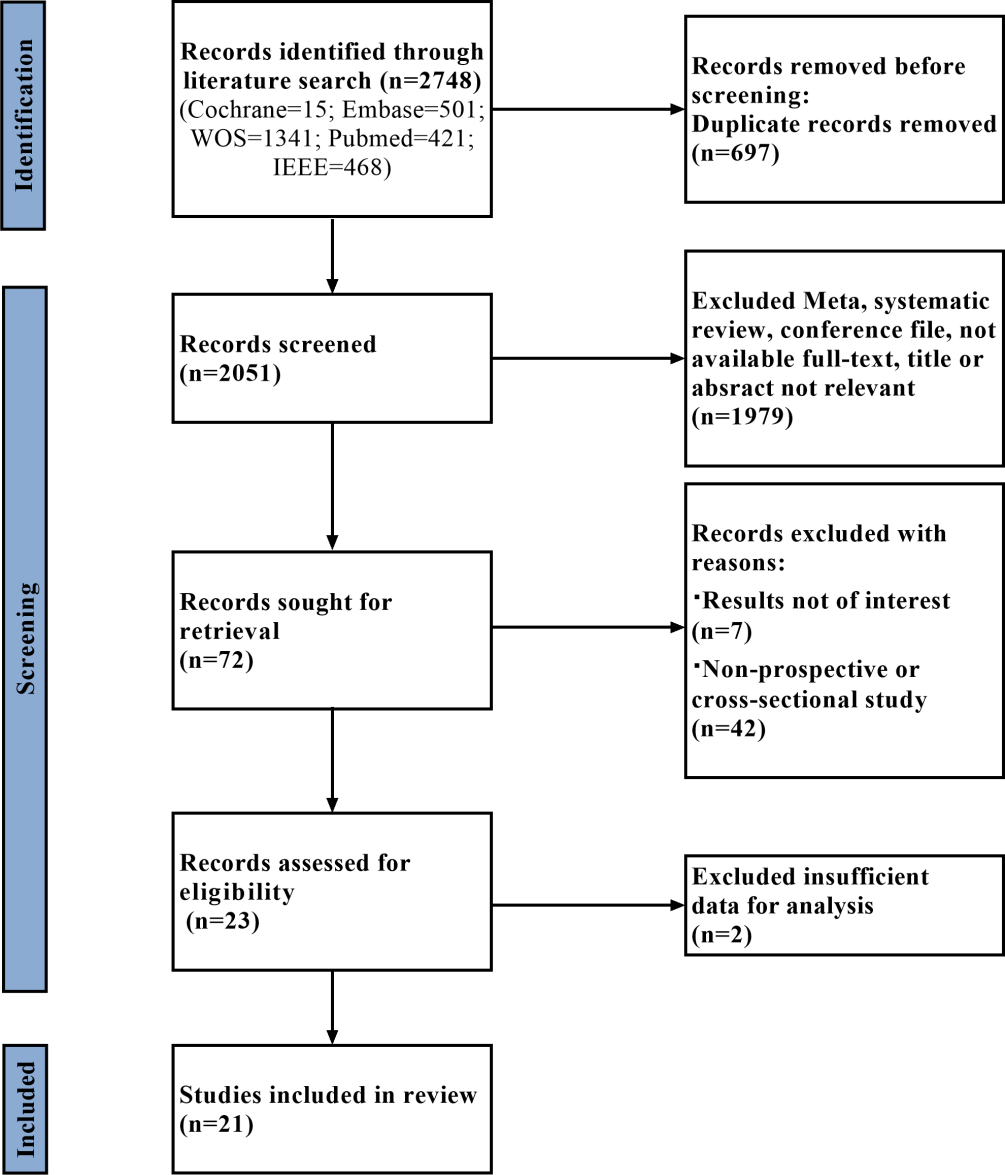
Flow diagram of literature selection.

A flowchart of the literature search and study selection process is presented in **Figure 1**. Firstly, relevant studies were retrieved successively from the relevant databases according to the retrieval strategy, which yielded 2748 studies in total. Thereafter, duplicate studies, meta-analyses, reviews, conference files, studies whose full text could not be obtained, and studies whose title and abstract were inconsistent with the research content were eliminated. After the preliminary screening, we obtained 72 original studies. Next, we excluded studies that were not of interest, studies that were not prospective or cross-sectional, or had incomplete data for meta-analysis. Finally, 21 studies were used for quantitative synthesis of the meta-analysis (18–38). **Table 1** summarizes the outcome variables included in the study. The population included in the study was selected from the real world from cross-sectional or prospective studies, thus avoiding bias due to case-control studies. Among them, seven, 17, five, and four studies evaluated any DR, referable DR (RDR), more-than-mild DR (mtmDR), and vision-threatening DR (VTDR), respectively. In addition, 19 studies included patients from the clinic, seven from the general community, and seven from the ordinary population. We explored the algorithm used by each study for diagnosing DR, image quality, region where the study was conducted, and sample size (**Table 1**). **Table 2** summarized additional data about the patients, such as sex, age, type of diabetes, diabetes duration, co-morbidities and soon. The study was registered in the PROSPERO (CRD42023389687).

**Table 1:** Summary of the data obtained from the included studies.

Author	Year	country	reference standard	Categories of DR	TP^a^	FP^b^	FN^c^	TN^d^	Sample size (eyes)	Source of patients	Quality of literature	pixels	algorithm
Baget-Bernaldiz et al. (18)	2021	Spain	four expert retina ophthalmologists	any DR	2310	11	49	11816	14186	Health Care Area	low	Not clear	convolutional neuronal network
				RDR	1453	10	50	12673	14186	Health Care Area	low	Not clear	convolutional neuronal network
Bode, B.W et al. (19)	2019	US	Wisconsin Reading Center	mtmDR	296	209	14	1182	1701	primary care and eye care facilities	high	Not clear	machine learning
				vtDR	60	178	3	1436	1677	primary care and eye care facilities	high	Not clear	machine learning
Do Rio et al. (20)	2022	UK	retinal specialist	any DR	556	2358	216	14076	17206	community	medium	Not clear	deep neural network
Gulshan et al. (21)	2019	India	retinal specialist	mtmDR	615	95	77	1118	1905	eye care centers	high	Not clear	neural network
				VTDR	418	141	11	1376	1946	eye care centers	high	Not clear	neural network
Li et al. (22)	2022	China	retinal specialist	RDR	147	44	9	947	1147	Shanghai General Hospital	medium	800*800	deep learning
Ming et al. (23)	2021	China	Two licensed ophthalmologists	RDR	19	5	5	292	321	community	low	Not clear	convolutional neural network
				any DR	30	6	6	279	321	community	low	Not clear	convolutional neural network
Natarajan et al. (24)	2019	India	a vitreoretinal resident and a vitreoretinal surgeon	RDR	23	60	0	311	394	Population-based	high	Not clear	neural network
				any DR	36	28	6	324	394	Population-based	high	Not clear	neural network
Noriega et al. (25)	2021	Mexico	3 retina specialists	RDR	48	5	2	45	100	Mexican ophthalmic hospital	low	224*224	deep learning
Pawar et al. (26)	2021	India	four ophthalmologists	RDR	47	14	0	150	211	rural community eye clinic	low	Not clear	deep learning
Rego et al. (27)	2021	Portugal	3 ophthalmologists	RDR	38	7	9	241	295	primary care offices	low	Not clear	convolutional neural network
Rogers et al. (28)	2020	Finland	a board of experts.	RDR	486	944	109	4213	5752	clinical-based	medium	4288*2848	deep learning
				VTDR	52	700	8	4992	5752	clinical-based	medium	4288*2848	deep learning
Sandhu et al. (29)	2020	US	clinical ophthalmologists	any DR	75	1	0	35	111	a single academic medical center	high	1024*1024	machine learning
				mtmDR	75	1	0	35	111	a single academic medical center	high	1024*1024	machine learning
Scheetz et al. (30)	2021	Australia	two retinal specialists	RDR	31	21	1	150	203	clinical-based	low	Not clear	deep learning
Sosale et al. (31)	2020	India	vitreoretinal specialist	RDR	121	23	1	152	297	population-based	low	750*1334	convolutional neural networks
				any DR	105	8	16	168	297	population-based	low	750*1334	convolutional neural networks
Sosale et al. (32)	2019	India	retina specialist	RDR	231	49	17	603	900	population-based	low	512*512	Convolutional Neural Networks
				any DR	210	29	42	619	900	population-based	low	512*512	Convolutional Neural Networks
Tang et al. (33)	2021	China	retina specialist	RDR	178	11	10	214	413	clinical-based	low	2600*2048	convolutional neural networks
Ting et al. (34)	2017	China	a retinal specialist(>10 years’ experience)	RDR	976	2929	102	31941	35948	community	high	Not clear	convolutional neural networks
				VTDR	514	3154	0	32280	35948	community	high	Not clear	convolutional neural networks
				mtmDR	298	4026	22	31602	35948	community	high	Not clear	convolutional neural networks
Wang et al. (35)	2021	China	three trained graders	RDR	192	291	6	2115	2604	Clinica-based	medium	299*299	convolutional neural networks
Wongchaisuwat et al. (36)	2021	Thailand	a retinal expert	RDR	18	77	3	884	982	Clinical-based	medium	Not clear	convolutional neural networks
			a retinal expert	RDR	21	104	2	547	674	Clinical-based	medium	Not clear	convolutional neural networks
Yao et al. (37)	2022	China	two senior ophthalmologists	mtmDR	42	26	9	45	121	Clinical-based	low	Not clear	regression tree algorithm
Zhang et al. (38)	2020	China	a panel of three experts	RDR	8265	2306	1657	28437	40665	diabetes centers	low	800*800	neural network

a TP, true positive;

b FP, false positive;

c FN, false negative;

d TN, true negative; DR, diabetic retinopathy; RDR, referable DR; mtmDR, more-than-mild DR; VTDR, vision-threatening DR.

**Table 2:** Summary of additional characteristics of the included studies.

Author	Year	Categories of DR	Sex (male%)	Age	Type of diabetes	Random blood sugar	Hemoglobin A1c	Diabetes duration	Co-morbidities		
									BP^a^	Obesity^b^	BMI^c^ (kg/m2)
Baget-Bernaldiz et al. (18)	2021	any DR	54.6	63.7 years (mean)	type 2 diabetes	NM^d^	NM	NM	NM	NM	NM
		RDR	54.6	63.7 years (mean)	NM	NM	NM	NM	NM	NM	NM
Bode, B.W et al. (19)	2019	mtmDR	50.1	<65 years, 75.1% >=65 years, 24.9%	type 2 diabetes	NM	>=9%	NM	NM	NM	NM
		vtDR	50.1	<65 years, 75.1% >=65 years, 24.9%	type 2 diabetes	NM	>=9%	>=5 years	NM	NM	NM
Do Rio et al. (20)	2022	any DR	47.9	57.7 years (mean)	type 2 diabetes	>=8.9%	NM	NM	NM	NM	NM
Gulshan et al. (21)	2019	mtmDR	58.1	56.6 years (mean)	NM	NM	NM	NM	NM	NM	NM
		VTDR	58.1	56.6 years (mean)	NM	NM	NM	NM	NM	NM	NM
Li et al. (22)	2022	RDR	68.4	50 yeas (mean)	NM	NM	8.67%	9.79 years (median)	NM	overweight	25.67
Ming et al. (23)	2021	RDR	45.7	69.3 years (mean)	NM	NM	NM	11.2 years (mean)	NM	NM	NM
		any DR	45.7	69.3 years (mean)	type 2 diabetes	NM	NM	11.2 years (mean)	NM	NM	NM
Natarajan et al. (24)	2019	RDR	48.4	51.3 years (mean)	NM	NM	NM	NM	NM	NM	NM
		any DR	48.4	51.3 years (mean)	NM	NM	NM	NM	NM	NM	NM
Noriega et al. (25)	2021	RDR	NM	NM	NM	NM	NM	NM	NM	NM	NM
Pawar et al. (26)	2021	RDR	51.45	52.84 years (mean)	NM	NM	NM	NM	NM	NM	NM
Rego et al. (27)	2021	RDR	NM	NM	NM	NM	NM	NM	NM	NM	NM
Rogers et al. (28)	2020	RDR	34.2	60 years (mean)	NM	NM	NM	NM	NM	NM	NM
		VTDR	34.2	60 years (mean)	NM	NM	NM	NM	NM	NM	NM
Sandhu et al. (29)	2020	any DR	52	20-82 years	NM	NM	NM	NM	NM	NM	NM
		mtmDR	52	20-82 years	NM	NM	NM	NM	NM	NM	NM
Scheetz et al. (30)	2021	RDR	50	56 years (median)	type 1 diabetes(34%) type 2 diabetes(65%)	NM	NM	13 years(median)	NM	NM	NM
Sosale et al. (31)	2020	RDR	58	55 years (mean)	type 1, 2 diabetes	NM	8%	11 years (mean)	NM	overweight	27
		any DR	58	55 years (mean)	type 1, 2 diabetes	NM	8%	11 years (mean)	NM	overweight	27
Sosale et al. (32)	2019	RDR	NM	NM	NM	NM	NM	NM	NM	NM	NM
		any DR	NM	NM	NM	NM	NM	NM	NM	NM	NM
Tang et al. (33)	2021	RDR	52.5	60.81 years (mean)	type 1 diabetes(6%) type 2 diabetes(94%)	NM	NM	11.84 years	NM	NM	NM
Ting et al. (34)	2017	RDR	51.02	60.16 years (mean)	NM	NM	7.54%	3.7 years (median)	132/73	overweight	27.22
		VTDR	51.02	60.16 years (mean)	NM	NM	7.54%	3.7 years (median)	132/73	overweight	27.22
		mtmDR	51.02	60.16 years (mean)	NM	NM	7.54%	3.7 years (median)	132/73	overweight	27.22
Wang et al. (35)	2021	RDR	58.5%	59.1 years (mean)	NM	Fast blood glucose: 5.60 ± 1.17mmol/L	NM	6.44 years (mean)	129/75	no obesity	24
Wongchaisuwat et al. (36)	2021	RDR	NM	NM	NM	NM	NM	NM	NM	NM	NM
		RDR	NM	NM	NM	NM	NM	NM	NM	NM	NM
Yao et al. (37)	2022	mtmDR	61.16%	56.64 years (mean)	NM	NM	8.73%	9 years (mean)	NM	NM	NM
Zhang et al. (38)	2020	RDR	58.25%	18-29 years,3.4% 30-39 years,9.3% 40-49 years,21.3% 50-59 years,35.0% 60-69 years,25.3% >=70 years,5.7%	NM	NM	<6.5%,9.3% (6.5-6.9)%,7.5% (7.0-7.9)%,15.3% (8.0-8.9)%,14% (9.0-9.9)%,12.2% >=10%,27.9%	<5 years,42.2% 5-10 years,17.8% 10-15 years,13.3% 15-20 years,6% >=20 years,4%	NM	NM	NM

a BP, blood pressure,(mmHg, systolic blood pressure/diastolic blood pressure);

b obesity, according to the BMI index conversion (BMI=18.5-25kg/m^2^, no obesity; BMI=25-30kg/m^2^, overweight; BMI=30-35kg/m^2^, mild obesity; BMI=35-4040kg/m^2^, moderate obesity; BMI>40kg/m^2^, severe obesity);

c BMI, Body Mass Index, weight/height^2^;

d NM, Not Mentioned.

### 3.2 Quality assessment

**Figure 2:**
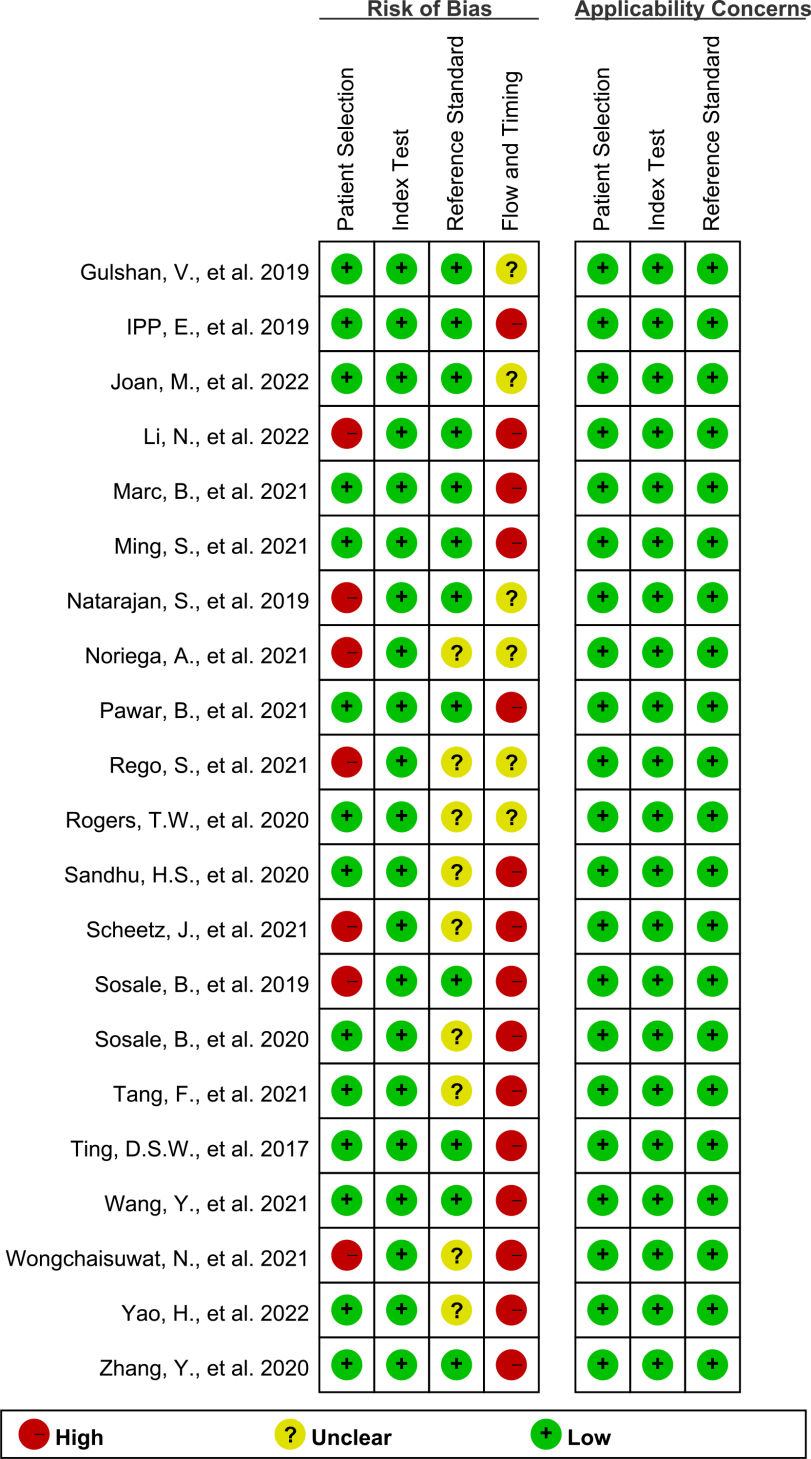
QUADAS-2 summary plot of bias risk assessment.

**Figure 3:**
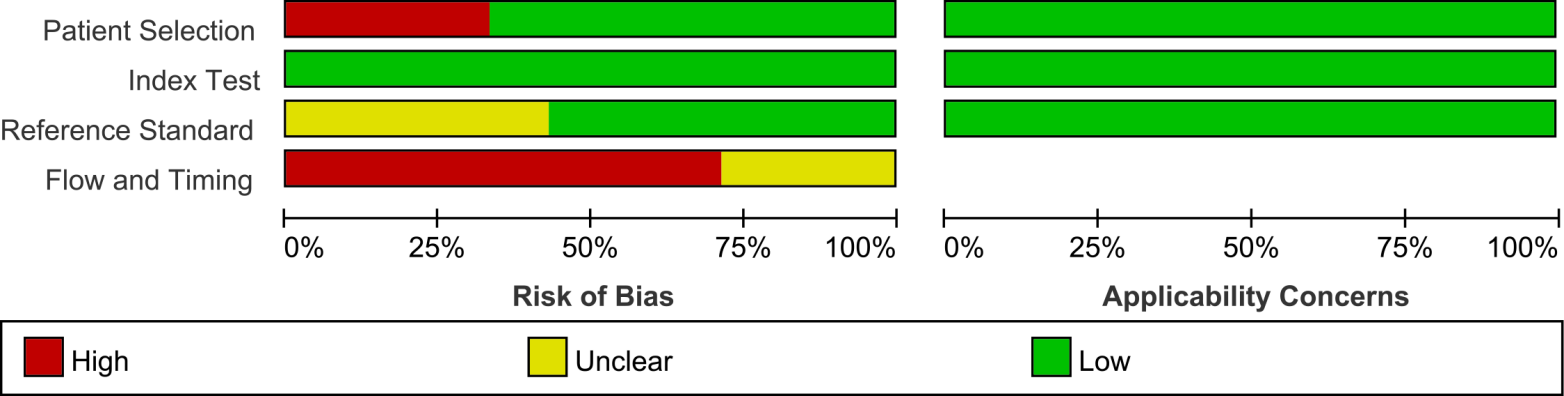
QUADAS-2 bar plot of bias risk assessment.

**Figures 2**, **3** show the summary chart and bar chart, respectively, for quality evaluation of the included studies, and **Supplementary Table S1** shows the process of quality evaluation using QUADAS-2. We found that seven studies answered no in patient selection, all studies performed well in the index test, and nine studies did not provide clear information for evaluating the reference standard. The included studies performed poorly in evaluating the flow and timing of patient selection; additionally, when evaluating patient selection, index test, and reference standard, all studies showed low risk with regard to clinical applicability concerns, indicating the high credibility of this meta-analysis.

### 3.3 Threshold analysis and heterogeneity test

The data were imported into MetaDiSc software (version 1.4) for analysis. It was found that the spearman correlation coefficient between the sensitivity logarithm and (1-specificity) logarithm was 0.001 (p=0.996>0.05), which was not significant. Therefore, there was no threshold effect (**Supplementary Table S2**) in this study. We then combined the DOR of all studies, and the Cochrane-Q test showed Cochrane-Q=1437.57, P=0.000<0.01, indicating that heterogeneity was caused by the non-threshold effect in this study. Moreover, the SE, SP, LR+, LR-, and DOR were all greater than 50% (**Table 3**). The above results show that heterogeneity existed between the studies, and may be related to the population, age, algorithm, and literature quality. Consequently, a random effects model (REM, DerSimonian-Laird method) was adopted to synthesize the above five indicators.

**Table 3:** The combined predictive value of all included studies.

Index	Merge value	95% Cis	I^2^ (%)	P value
Se^a^	0.880	0.875-0.884	97.3	0.00
Sp^b^	0.912	0.911-0.913	99.5	0.00
DOR^c^	206.80	124.82-342.63	97.8	0.00
LR+^d^	13.021	10.738-15.789	99.0	0.00
LR-^e^	0.083	0.061-0.112	96.5	0.00

a Se, sensitivity;

b Sp, specificity;

c DOR, diagnostic odds ratio;

d LR+, positive likelihood ratio;

e LR-, negative likelihood ratio.

### 3.4 Synthesis of results

**Figure 4:**
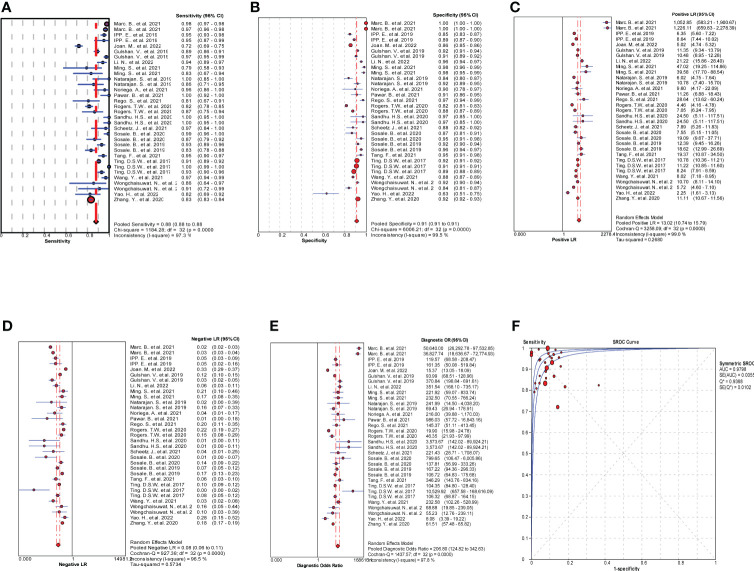
Results of meta-analysis and forest plots of all the included studies. **(A)** Forest plot of pooled Se. **(B)** Forest plot of pooled Sp. **(C)** Forest plot of pooled positive likelihood ratio (LR+). **(D)** Forest plot of pooled negative likelihood ratio (LR-). **(E)** Forest plot of pooled diagnostic odds ratio (DOR). **(F)** Summary receiver operating characteristic (SROC) plots.

**Figure 5:**
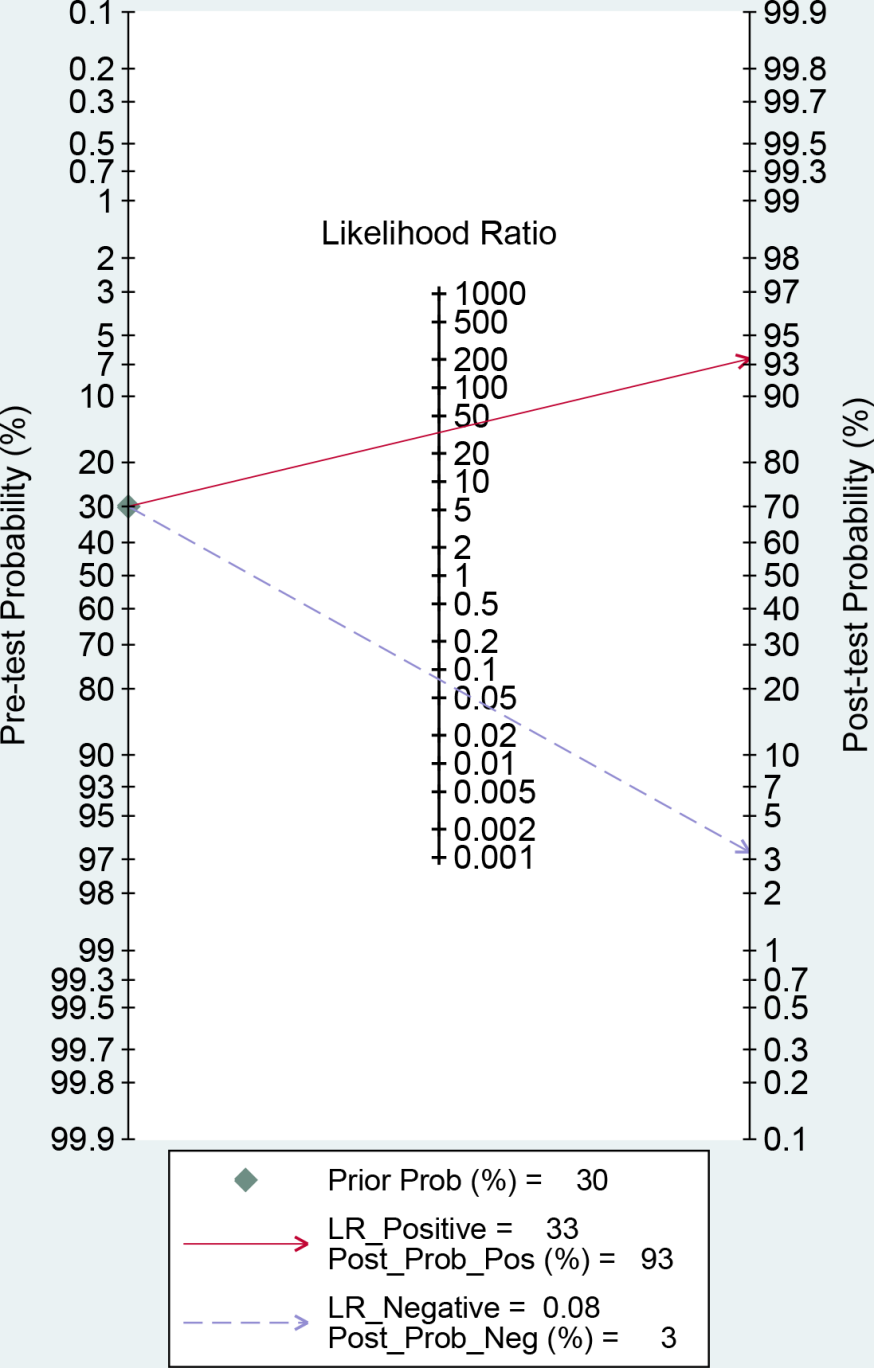
Fagan nomogram of artificial intelligence (AI) for the diagnosis of any diabetic retinopathy (DR).

MetaDiSc software was used to analyze the included data. The pooled SE was 0.880 (0.875-0.884), pooled SP was 0.912 (0.99-0.913), pooled LR+ was 13.021 (10.738-15.789), pooled LR- was 0.083 (0.061-0.112), combined AUC=0.9798, Q index =0.9388, and pooled DOR was 206.80 (124.82-342.63). Corresponding (SROC) plots and coupled forest plots are shown in **Figure 4**; **Table 2**, respectively. For further analyzing the diagnostic efficacy of AI in diagnosing any DR, 0.3 was set as the pretest probability. On drawing fagan nomogram (**Figure 5**), it was found that the positive post-test probability was 93% and negative post-test probability was 3%. Next, we grouped all included studies in accordance with categories of DR (any DR/RDR/mtmDR/VTDR), patient source (clinical-based/community-based/population-based), country (non-Asia/Asia), sample size (<5000 eyes/>5000 eyes), quality of literature (low quality/medium quality/high quality), image pixels (<1000*1000/>1000*1000), algorithm (convolutional neural network/machine learning/neural network/others [deep learning, regression tree algorithm]), and performed subgroup analysis. The results are shown in **Table 4**, and the SROC plot of each subgroup is shown in **Supplementary Figures S1**-**S7**.

**Table 4:** Results of subgroup analysis.

Subgroup	Number of study	[pooled Se (95%CI)/I^2^]	[pooled Sp (95%CI)/I^2^]	AUC	[DOR (95%CI)/I^2^]	[RDOR^a^ (95%CI)/P value]
**Categories of DR**						0.68 (0.23-2.02)/0.4747
any DR	7	0.91 (0.900-0.919)/98.7	0.917 (0.914-0.920)/99.8	0.9846	400.16 (23.709-6754.0)/99.2	
RDR	17	0.861 (0.855-0.867)/96.0	0.924 (0.923-0.926)/99.4	0.9780	205.59 (110.14-383.76)/97.1	
mtmDR	5	0.916 (0.900-0.930)/87.5	0.886 (0.883-0.890)/94.1	0.9417	74.829 (33.069-169.32)/89.0	
VTDR	4	0.979 (0.969-0.987)/92.6	0.906 (0.903-0.908)/95.5	0.9446	299.16 (48.362-1850.6)/92.4	
**Source of patient**						0.69 (0.22-2.13)/0.5044
clinical-based	19	0.879 (0.873-0.883)/97.8	0.935 (0.933-0.937)/99.6	0.9804	246.49 (108.49-560.05)/98.3	
community-based	7	0.874 (0.861-0.886)/98.0	0.899 (0.897-0.900)/99.0	0.9778	171.47 (53.469-549.90)/98.0	
population-based	7	0.913 (0.893-0.929)/88.4	0.898 (0.889-0.906)/91.0	0.9738	144.06 (96.850-214.29)/31.9	
**Country**						0.33 (0.06-1.80)/0.1906
non-Asia	12	0.922 (0.915-0.929)/98.1	0.920 (0.917-0.922)/99.8	0.9854	399.01 (78.607-2025.3)/99.1	
Asia	21	0.862 (0.856-0.868)/95.5	0.909 (0.908-0.911)/96.4	0.9721	135.23 (96.122-190.26)/88.7	
**Sample size**						2.58 (0.46;14.61)/0.2697
<5000 eyes	18	0.913 (0.897-0.927)/82.8	0.915 (0.907-0.922)/91.5	0.9744	151.75 (87.790-262.30)/73.7	
>5000 eyes	15	0.877 (0.872-0.882)/98.7	0.912 (0.911-0.913)/99.8	0.9828	253.11 (121.86-525.71)/99.0	
**Quality of included studies**						0.63 (0.22-1.87)/0.3925
low	15	0.875 (0.870-0.880)/98.1	0.957 (0.955-0.958)/99.5	0.9815	356.42 (96.015-1323.0)/98.4	
medium	7	0.807 (0.788-0.824)/94.6	0.860 (0.856-0.864)/97.4	0.9487	60.018 (28.850-124.86)/94.9	
high	11	0.935 (0.927-0.943)/93.2	0.904 (0.902-0.905)/96.2	0.9850	157.27 (100.48-246.13)/78.7	
**Pixels of image**						0.83 (0.33-2.10)/0.6860
<1000*1000	6	0.840 (0.833-0.847)/93.8	0.923 (0.920-0.926)/94.2	0.9740	151.90 (78.281-294.76)/89.6	
>1000*1000	7	0.883 (0.864-0.901)/93.5	0.853 (0.847-0.860)/95.4	0.9703	207.04 (55.647-770.29)/93.0	
**Algorithm**						0.42 (0.23-0.77)/0.0072
CNN^b^	16	0.952 (0.947-0.957)/94.0	0.922 (0.920-0.923)/99.7	0.9862	402.96 (136.89-1184.6)/97.6	
ML^c^	4	0.967 (0.948-0.981)/74.4	0.874 (0.861-0.885)/84.7	0.7278	289.92 (82.140-1023.3)/62.8	
NN^d^	5	0.842 (0.835-0.849)/96.3	0.923 (0.920-0.926)/88.9	0.9685	111.69 (60.069-207.67)/89.7	
others^e^	8	0.799 (0.780-0.818)/92.8	0.857 (0.853-0.861)/96.7	0.9366	50.616 (25.408-100.83)/92.9	

a RDOR, relative diagnostic odds ratio;

b CNN, convolutional neural network;

c ML, machine learning;

d NN, neural network;

e others, deep learning, regression tree algorithm.

### 3.5 Meta regression and sensitivity analysis

To explore the source of heterogeneity, we performed meta regression according to the conditions of subgroup analysis using MetaDiSc software. We found that the p value of the algorithm term was 0.033<0.05, indicating that when different AI models are uses to diagnose DR, the algorithms used by the different models may be the source of heterogeneity. The results are shown in **Table 5**. STATA 14.0 was selected for conducting the sensitivity analysis. It can be clearly seen in **Supplementary Figure S9** that there were three original studies with strong sensitivity and the results did not differ significantly. The deleted results are shown in **Supplementary Figure S8** and **Supplementary Table S3**. Other original studies did not demonstrate obvious sensitivity. Overall, the results of our study were stable.

**Table 5:** Meta regression of included studies.

Var	Coeff.	Std. Err.	p value	RDOR	[95%CI]
Cte.	9.872	2.0250	0.0001	—	—
S	0.300	0.2752	0.2861	—	—
categories^a^	-0.382	0.5265	0.4747	0.68	(0.23;2.02)
source^b^	-0.370	0.5465	0.5044	0.69	(0.22;2.13)
country^c^	-1.106	0.8214	0.1906	0.33	(0.06;1.80)
quality^d^	-0.456	0.5233	0.3925	0.63	(0.22;1.87)
pixels^e^	-0.183	0.4477	0.6860	0.83	(0.33;2.10)
algorithm^f^	-0.861	0.2933	0.0072	0.42	(0.23;0.77)
sample^g^	0.949	0.8396	0.2697	2.58	(0.46;14.61)

Tau-squared estimate = 3.0652 (Convergence is achieved after 7 iterations).

a categories, categories of diabetic retinopathy (any DR/RDR/mtmDR/VTDR).

b source, source of patient (clinical-based/community-based/population-based).

c country, region where the study was conducted (non-Asia/Asia).

d quality, quality of included studies (low/medium/high).

e pixels, pixels of fundus color photograph (<1000*1000/>1000*1000).

f algorithm, algorithm of applied artificial intelligence model (convolutional neural network/machine learning/neural network/others [deep learning, regression tree algorithm]).

g sample, sample size of included studies (<5000 eyes/>5000 eyes).

“—”, null value.

### 3.6 Publication bias

STATA 14.0 was used to first conduct a publication bias test for all data included in the study, followed by a publication bias test for different categories of DR. The results are shown in **Supplementary Figure S10**. For all the included studies, p value was 0.84>0.05, which means that there was no publication bias in this study, based on the symmetry of the funnel plot.

## 4 Discussion

In recent years, several studies have investigated the use of AI for the assessment of incidence and diagnostic accuracy of DR. Meanwhile, the application of AI in the medical field is being continuously developed and subdivided (39). However, due to the different image quality of fundus color photos, algorithms used by AI models, and performance of cameras used in different studies, the AI diagnostic effect varies significantly among different studies (40, 41). Our study is the first meta-analysis to include all prospective studies and use multiple algorithms to evaluate the diagnostic efficacy in DR, rather than only deep learning or machine learning. Firstly, relevant studies were retrieved from medical databases according to the retrieval strategy, and the retrieved studies was screened according to the existing guidelines for diagnostic reviews (42) to ensure the rigor of the study. In the final meta-analysis, we included a total of 21 original studies, involving 129,759 eyes. All studies were conducted in the real world, avoiding the bias caused by retrospective studies.

There was no threshold effect in this analysis, but the heterogeneity among studies was high. Therefore, the random effects model was used to combine all indicators. DOR and AUC were the main indicators to judge the relationship between the diagnostic results and DR. The pooled DOR=206.80 and AUC=0.9798 for all included studies indicated that AI had a high diagnostic performance for DR. In order to make the study results more clinically relevant, we drew fagan plots and concluded that if AI showed a positive result based on fundus color photography, the probability of the patient having DR was 93%. If the AI diagnosis was negative, the patient had a 3% chance of DR.

To explore the source of heterogeneity, we conducted meta regression, and found that the differences in AI algorithms may be the source of heterogeneity. To further explore the factors influencing the AI diagnosis of DR, we performed a subgroup analysis. We found that studies with patients from clinics, hospitals, or medical research centers had higher diagnostic efficacy than those with patients from other sources, and this may be because patients from hospitals or medical research centers are more representative, and retinopathy can be diagnosed more accurately by clinicians with a lower error rate; besides studies from non-Asian countries had higher diagnostic efficacy than studies from Asian countries, and we believed that this is because non-Asian countries have carried out artificial intelligence algorithm diagnosis DR for a long time, trained the algorithm more times, had a large data set, included more cases, and had relatively high data quality. Moreover, we have found that the greater the number of eyes included in the study, the higher the diagnostic efficacy. We hypothesized that the more cases included, the more times the algorithm would be trained, so the more accurate the diagnosis would be. When the image pixel was taken as the standard to judge the image quality, it was found that the higher the image quality, the higher the DOR value of the diagnostic result, which is similar to the findings of Yip et al. (43). When the algorithm was taken as a subgroup for analysis, the diagnostic effectiveness of the convolutional neural network (CNN) algorithm was significantly higher than that of other algorithms. CNN is the most widely used in the field of medical imaging, which approximates the work efficiency and reliability of experienced clinicians (44). We also found that when the included studies were of high quality, the heterogeneity was significantly lower than when studies were of lower quality. The high diagnostic performance of the above results maybe the result of the large number and high representativeness of the included studies. This may be because high-quality research used more training of AI models, and clinical trials are better arranged, scientific, and include more representative cases, so the results are more reliable.

Several studies have found that for screening, risk stratification, management, and prognosis of DR, the effect of AI cannot be ignored. Firstly, an AI-based automated system can improve the efficiency and coverage of DR diagnosis and treatment, since the traditional DR diagnosis and treatment process only relies on a pattern of manual identification, which is easily affected by the experience, skills, and other factors related to the ophthalmologist or relevant technical personnel; therefore, the efficiency of DR screening is relatively low (45). Secondly, DR Patients in remote areas can miss the opportunity to undergo timely treatment due to the lack of skilled ophthalmologists (46). The application of AI in telemedicine can solve this problem (41). Thirdly, it can help clinicians to develop appropriate treatment strategies based on the individual disease of the patients. Clinically, proliferative diabetic retinopathy (PDR) patients are usually treated with laser, intravitreous injection of anti-vascular endothelial growth factor (VEGF), or corticosteroid drugs (47). If the above treatment is given at an early stage to patients with non-proliferative diabetic retinopathy (NPDR) due to an incorrect diagnosis, it will not only waste medical resources, but may also cause serious complications (48). Moreover, studies have shown that AI-based DR screening is more cost-effective than manual grading, and may help in providing cost-effective, convenient, and effective medical services (49).

As a novel diagnostic tool, there are still many problems with AI: (1) Although AI is getting better at diagnosing eye diseases, in our study, the false negative rate (FNR) was 12% and false positive rate FPR was 8.8%, which cannot be ignored. Further exploration of imaging features, increasing the sample size of the training set and test set, or further improving the performance of the algorithm are all feasible methods to solve these problems (43). (2) At present, the models established by various AI algorithms are still considered “black box”. This model lacks “explanatory ability” for the diagnosed diseases, that is, it cannot provide the reasons for the diagnostic results to clinicians (50, 51). (3) Since most current studies have detected DR through fundus imaging, the results may not be applicable to other eye diseases and imaging methods.

The ophthalmologist will play an important role in judging the clinical value of emerging AI technologies, in addition to a guiding role in integrating complementary imaging information with clinical data to provide more complete diagnostic information (52). Even if AI can diagnose DR independently, the ophthalmologist will eventually have to issue a report and take legal risks (53); therefore, legislation is required to clarify the respective scope of responsibility between doctors and companies providing AI services, which may also promote the popularization of AI diagnostic services. What is satisfactory is that currently both doctors and patients have a positive attitude towards the diagnostic efficacy of AI (54, 55), which may lay a foundation for their subsequent cooperation.

This study has the following limitations: (1) the collected DR data lacked proliferative diabetic subtype or further classification of DR, which may affect the evaluation of the diagnostic value; (2) some studies lacked four-grid table data or contained a small number of samples; therefore, the diagnostic value of the representative algorithm may not be truly reflected; (3) In the meta regression, we did not further analyze patient information, such as age, sex, and duration of the disease, which may be a source of heterogeneity and need further study; (4) only English studies were included, which may cause a bias due to the lack of literature in other languages; (5) The gold standard is the decision made by an ophthalmologist or retinologist based on the fundus color image, which means that AI may not perform well on images that an ophthalmologist cannot recognize; (6) Most of the AI algorithm models used in studies are self-developed or debugged models. Since the researchers did not clarify the pre-training degree and learning amount of each model, we could not include these factors in the analysis; (7) there are problems with direct comparison of diagnostic accuracy. As can be seen from the high diagnostic accuracy of VTDR, the diagnostic accuracy differs between mild retinopathy and severe retinopathy. Therefore, the overall accuracy will change depending on the composition ratio of the disease stage of the image for accuracy verification. For example, if many of the accuracy verification images are of mild retinopathy, it is difficult to recognize the lesions, so false negatives increase and accuracy is predicted to decrease.

In conclusion, this meta-analysis suggests that AI-based fundus color imaging has a high predictive ability for DR. The diagnosis rate is much higher than the manual, method, which can contribute to the clinical development of the follow-up strategy or diagnosis and treatment plan and has a high practical application value. However, AI still has a certain rate of missed diagnosis and misdiagnosis, and is easily affected by the patient source, number and representativeness of sample, algorithm of the AI model, quality of images, use of cameras, and type of algorithm. Correspondingly, the performance of AI for diagnosis of DR can be further improved by obtaining more detailed patient data, collecting a large number of samples from multi-centers, deep mining of image features, optimizing AI algorithm architecture, and using high-resolution cameras for images. If the diagnosis and treatment strategies formulated by ophthalmologists are combined with AI, the work efficiency can be greatly improved and the utilization rate of medical resources can be increased, in addition to providing a more scientific and efficient way for early screening, diagnosis, and treatment of DR.

## Data availability statement

The original contributions presented in the study are included in the article/**Supplementary Material**. Further inquiries can be directed to the corresponding author.

## Author contributions

KL and SM were responsible for searching the studies in the databases. ZW and ZL designed the entire research idea, selected the appropriate studies after careful searching, and extracted the data from each study. SM and XZ completed the quality assessment. Final meta-analysis was completed by ZW. XZ and YD conceived the study and wrote the first draft. The four authors jointly revised the manuscript. All authors contributed to the article and approved the submitted version.
