# Supplementary material for: Performance of artificial intelligence in diabetic retinopathy screening: a systematic review and meta-analysis of prospective studies
Source: Front Endocrinol (Lausanne). 2023 Jun 13;14:1197783. doi: 10.3389/fendo.2023.1197783 (PMC10296189; doi:10.3389/fendo.2023.1197783)
Supplement: Supplementary file 2 [file DataSheet_2.doc]

Supplementary Table S1 Evaluation of risk bias in included studies

| study | Patient Selection | | | Index Test | | Reference Standard | | Flow and Timing | | | Applicability Concerns | | |
| --- | --- | --- | --- | --- | --- | --- | --- | --- | --- | --- | --- | --- | --- |
|  | continuous or random cases were included | case-control study designs were avoided | Study whether inappropriate exclusions are avoided | Whether the results of the trial to be evaluated were interpreted without knowing the results of the gold standard experiment | If a threshold is used, whether it is pre-researched | Whether the gold standard correctly identifies the target disease state | The gold standard result determines whether the blind method was used | The appropriate time interval between the experiment to be evaluated and the gold standard | Whether all patients received the same gold standard | Whether all cases were included in the analysis | Patient Selection | Index Test | Reference Standard |
| Baget-Bernaldiz., et al. 2021 | yes | yes | yes | yes | yes | yes | yes | not clear | yes | no | low | low | low |
| Bode, B.W., et al. 2019 | yes | yes | yes | yes | yes | yes | yes | not clear | yes | no | low | low | low |
| Do Rio., et al. 2022 | yes | yes | yes | yes | yes | yes | yes | not clear | yes | yes | low | low | low |
| Gulshan., et al. 2019 | yes | yes | yes | yes | yes | yes | yes | not clear | yes | yes | low | low | low |
| Li, N., et al. 2022 | yes | no | yes | yes | yes | yes | yes | not clear | yes | no | low | low | low |
| Ming, S., et al. 2021 | yes | yes | yes | yes | yes | yes | yes | not clear | yes | no | low | low | low |
| Natarajan, S., et al. 2019 | yes | no | yes | yes | yes | yes | yes | not clear | yes | yes | low | low | low |
| Noriega, A., et al. 2021 | yes | no | yes | yes | yes | yes | not clear | not clear | yes | yes | low | low | low |
| Pawar, B., et al. 2021 | yes | yes | yes | yes | yes | yes | yes | not clear | yes | no | low | low | low |
| Rego, S., et al. 2021 | yes | no | yes | yes | yes | yes | not clear | not clear | yes | yes | low | low | low |
| Rogers, T.W., et al. 2020 | yes | yes | yes | yes | yes | yes | not clear | not clear | yes | yes | low | low | low |
| Sandhu, H.S., et al. 2020 | yes | yes | yes | yes | yes | yes | not clear | not clear | yes | no | low | low | low |
| Scheetz, J., et al. 2021 | yes | no | yes | yes | yes | yes | not clear | not clear | yes | no | low | low | low |
| Sosale, B., et al. 2020 | yes | yes | yes | yes | yes | yes | not clear | not clear | yes | no | low | low | low |
| Sosale, B., et al. 2019 | yes | no | yes | yes | yes | yes | yes | not clear | yes | no | low | low | low |
| Tang, F., et al. 2021 | yes | yes | yes | yes | yes | yes | not clear | not clear | yes | no | low | low | low |
| Ting, D.S.W., et al. 2017 | yes | yes | yes | yes | yes | yes | yes | not clear | yes | no | low | low | low |
| Wang, Y., et al. 2021 | yes | yes | yes | yes | yes | yes | yes | not clear | yes | no | low | low | low |
| Wongchaisuwat, N., et al. 2021 | yes | no | yes | yes | yes | yes | not clear | not clear | yes | no | low | low | low |
| Yao, H., et al. 2022 | yes | yes | yes | yes | yes | yes | not clear | not clear | yes | no | low | low | low |
| Zhang, Y., et al. 2020 | yes | yes | yes | yes | yes | yes | yes | not clear | yes | no | low | low | low |

Supplementary Table S2 Threshold analysis of included study using MetaDiSc 1.4

Spearman correlation coefficient: 0.001 p-value= 0.996

(Logit(TPR) vs Logit(FPR)

Moses' model (D = a + bS)

Weighted regression (Inverse Variance)

Var Coeff. Std. Error T p-value

a 5.393 0.346 15.589 0.0000

b( 1) -0.120 0.203 0.589 0.5600

Tau-squared estimate = 3.4961 (Convergence is achieved after 6 iterations)

Restricted Maximum Likelihood estimation (REML)

No. studies = 33

Supplementary Table S3 The combined predictive value after 3 sensitive studies were excluded

| **Index** | **Merge value** | **95% CIs** | **I^2^ (%)** | **P value** |
| --- | --- | --- | --- | --- |
| Se | 0.853 | 0.847-0.858 | 94.0 | 0.00 |
| Sp | 0.897 | 0.896-0.899 | 97.9 | 0.00 |
| DOR | 114.74 | 79.934-164.71 | 94.9 | 0.00 |
| LR+ | 9.741 | 8.321-11.404 | 97.9 | 0.00 |
| LR- | 0.105 | 0.084-0.131 | 92.0 | 0.00 |

Supplementary Table S4 PRISMA2020 checklist

| **Section and Topic** | **Item #** | **Checklist item** | **Location where item is reported** |
| --- | --- | --- | --- |
| **TITLE** | | |  |
| Title | 1 | Identify the report as a systematic review. | Page1. See title |
| **ABSTRACT** | | |  |
| Abstract | 2 | See the PRISMA 2020 for Abstracts checklist. | Page1. See line 10-32 |
| **INTRODUCTION** | | |  |
| Rationale | 3 | Describe the rationale for the review in the context of existing knowledge. | Page 2-3. See line 10-32 |
| Objectives | 4 | Provide an explicit statement of the objective(s) or question(s) the review addresses. | Page 3. See line 74-80 |
| **METHODS** | | |  |
| Eligibility criteria | 5 | Specify the inclusion and exclusion criteria for the review and how studies were grouped for the syntheses. | Page 3. See line 98-108 |
| Information sources | 6 | Specify all databases, registers, websites, organisations, reference lists and other sources searched or consulted to identify studies. Specify the date when each source was last searched or consulted. | **√** |
| Search strategy | 7 | Present the full search strategies for all databases, registers and websites, including any filters and limits used. | Page 3. See line 86-96 |
| Selection process | 8 | Specify the methods used to decide whether a study met the inclusion criteria of the review, including how many reviewers screened each record and each report retrieved, whether they worked independently, and if applicable, details of automation tools used in the process. | Page 9. See line 345-346 |
| Data collection process | 9 | Specify the methods used to collect data from reports, including how many reviewers collected data from each report, whether they worked independently, any processes for obtaining or confirming data from study investigators, and if applicable, details of automation tools used in the process. | Page 9. See line 346-347 |
| Data items | 10a | List and define all outcomes for which data were sought. Specify whether all results that were compatible with each outcome domain in each study were sought (e.g. for all measures, time points, analyses), and if not, the methods used to decide which results to collect. | file “original data.xlsx” |
|  | 10b | List and define all other variables for which data were sought (e.g. participant and intervention characteristics, funding sources). Describe any assumptions made about any missing or unclear information. | file “original data.xlsx” |
| Study risk of bias assessment | 11 | Specify the methods used to assess risk of bias in the included studies, including details of the tool(s) used, how many reviewers assessed each study and whether they worked independently, and if applicable, details of automation tools used in the process. | Page 4. See line 149-151 |
| Effect measures | 12 | Specify for each outcome the effect measure(s) (e.g. risk ratio, mean difference) used in the synthesis or presentation of results. | Page 6. See line 195-198 |
| Synthesis methods | 13a | Describe the processes used to decide which studies were eligible for each synthesis (e.g. tabulating the study intervention characteristics and comparing against the planned groups for each synthesis (item #5)). | Page 17. See table 1 |
|  | 13b | Describe any methods required to prepare the data for presentation or synthesis, such as handling of missing summary statistics, or data conversions. | Page 4. See line 132-151 |
|  | 13c | Describe any methods used to tabulate or visually display results of individual studies and syntheses. | Page 4. See line 132-151 |
|  | 13d | Describe any methods used to synthesize results and provide a rationale for the choice(s). If meta-analysis was performed, describe the model(s), method(s) to identify the presence and extent of statistical heterogeneity, and software package(s) used. | Page 4. See line 132-151 |
|  | 13e | Describe any methods used to explore possible causes of heterogeneity among study results (e.g. subgroup analysis, meta-regression). | Page 4. See line 132-151. Table 3,4 |
|  | 13f | Describe any sensitivity analyses conducted to assess robustness of the synthesized results. | Page 4. See line 132-151 |
| Reporting bias assessment | 14 | Describe any methods used to assess risk of bias due to missing results in a synthesis (arising from reporting biases). | Page 4. See line 148-151 |
| Certainty assessment | 15 | Describe any methods used to assess certainty (or confidence) in the body of evidence for an outcome. | Page 4. See line 132-151 |
| **RESULTS** | | |  |
| Study selection | 16a | Describe the results of the search and selection process, from the number of records identified in the search to the number of studies included in the review, ideally using a flow diagram. | Page 5. See line 154-171 |
|  | 16b | Cite studies that might appear to meet the inclusion criteria, but which were excluded, and explain why they were excluded. | Figure 1 |
| Study characteristics | 17 | Cite each included study and present its characteristics. | Table 1 |
| Risk of bias in studies | 18 | Present assessments of risk of bias for each included study. | Figure 2, 3 |
| Results of individual studies | 19 | For all outcomes, present, for each study: (a) summary statistics for each group (where appropriate) and (b) an effect estimate and its precision (e.g. confidence/credible interval), ideally using structured tables or plots. | Table 2 |
| Results of syntheses | 20a | For each synthesis, briefly summarise the characteristics and risk of bias among contributing studies. | file “original data.xlsx” |
|  | 20b | Present results of all statistical syntheses conducted. If meta-analysis was done, present for each the summary estimate and its precision (e.g. confidence/credible interval) and measures of statistical heterogeneity. If comparing groups, describe the direction of the effect. | Table 1, 2 |
|  | 20c | Present results of all investigations of possible causes of heterogeneity among study results. | Table 3 |
|  | 20d | Present results of all sensitivity analyses conducted to assess the robustness of the synthesized results. | Supplementary Figure S9 |
| Reporting biases | 21 | Present assessments of risk of bias due to missing results (arising from reporting biases) for each synthesis assessed. | Supplementary Figure S10 |
| Certainty of evidence | 22 | Present assessments of certainty (or confidence) in the body of evidence for each outcome assessed. | Table 2 |
| **DISCUSSION** | | |  |
| Discussion | 23a | Provide a general interpretation of the results in the context of other evidence. | Page 6, 7.See line 234-268 |
|  | 23b | Discuss any limitations of the evidence included in the review. | Page 8, 9.See line 307-326 |
|  | 23c | Discuss any limitations of the review processes used. | Page 8, 9.See line 307-326 |
|  | 23d | Discuss implications of the results for practice, policy, and future research. | Page 9. See line 327-341 |
| **OTHER INFORMATION** | | |  |
| Registration and protocol | 24a | Provide registration information for the review, including register name and registration number, or state that the review was not registered. | Page 1. See line 23 |
|  | 24b | Indicate where the review protocol can be accessed, or state that a protocol was not prepared. | protocol was not prepared. |
|  | 24c | Describe and explain any amendments to information provided at registration or in the protocol. | Not applicable. |
| Support | 25 | Describe sources of financial or non-financial support for the review, and the role of the funders or sponsors in the review. | Page 9. See line 348-351. |
| Competing interests | 26 | Declare any competing interests of review authors. | Not applicable. |
| Availability of data, code and other materials | 27 | Report which of the following are publicly available and where they can be found: template data collection forms; data extracted from included studies; data used for all analyses; analytic code; any other materials used in the review. | References to these information can be found in the original manuscript and supplementary materials. |


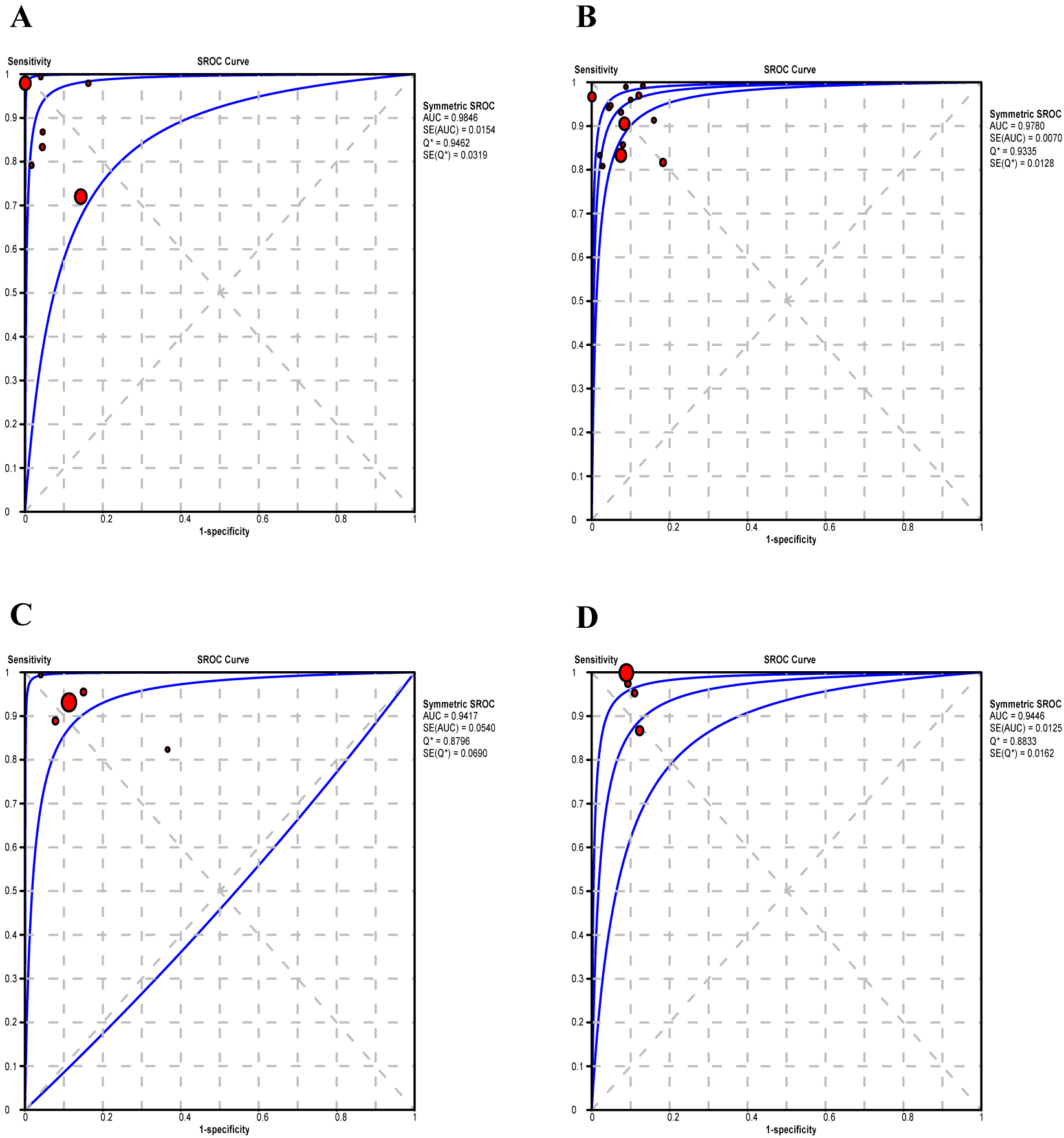


**Supplementary Figure S1**. Summary receiver operating characteristic (SROC) curves for the subgroup of categories of diabetic retinopathy (DR). **(A)** Any DR. **(B)** Referable DR (RDR). **(C)** More-than-mild DR (mtmDR). **(D)** Vision-threatening DR (VTDR)


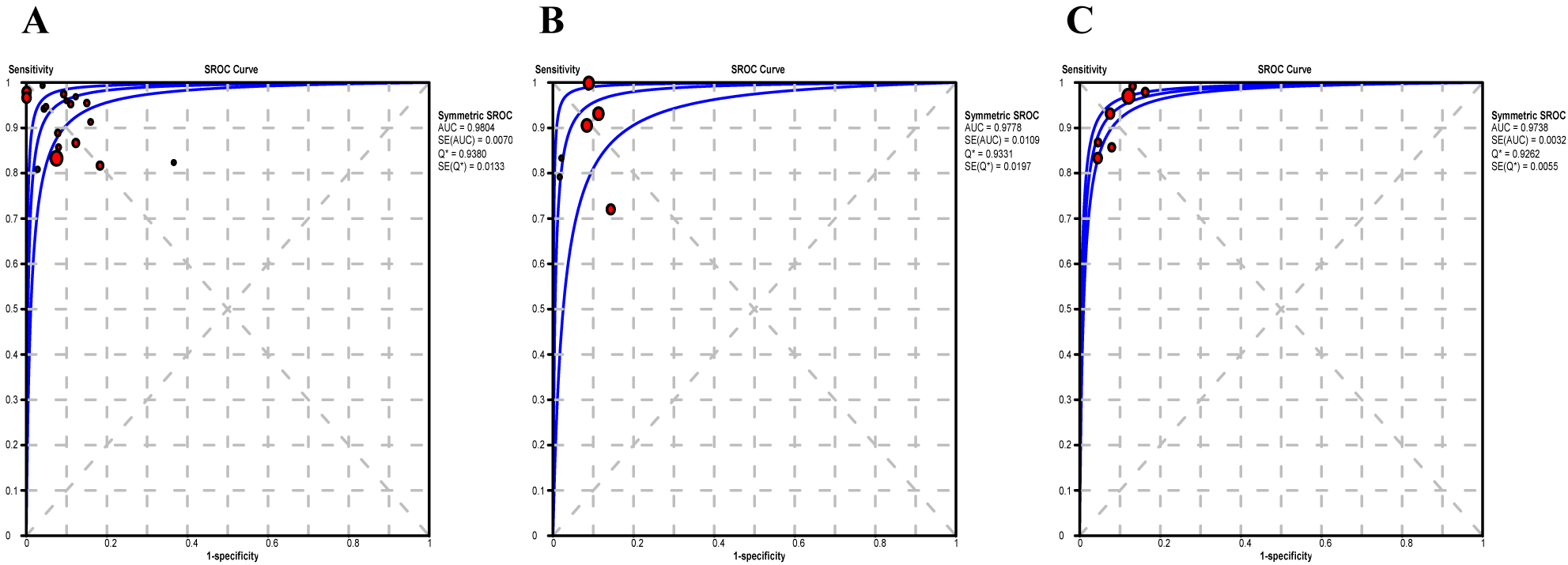


**Supplementary Figure S2**. Summary receiver operating characteristic (SROC) curves for the source of patients subgroup. **(A)** Clinical-based. **(B)** Community-based. **(C)** Population-based.


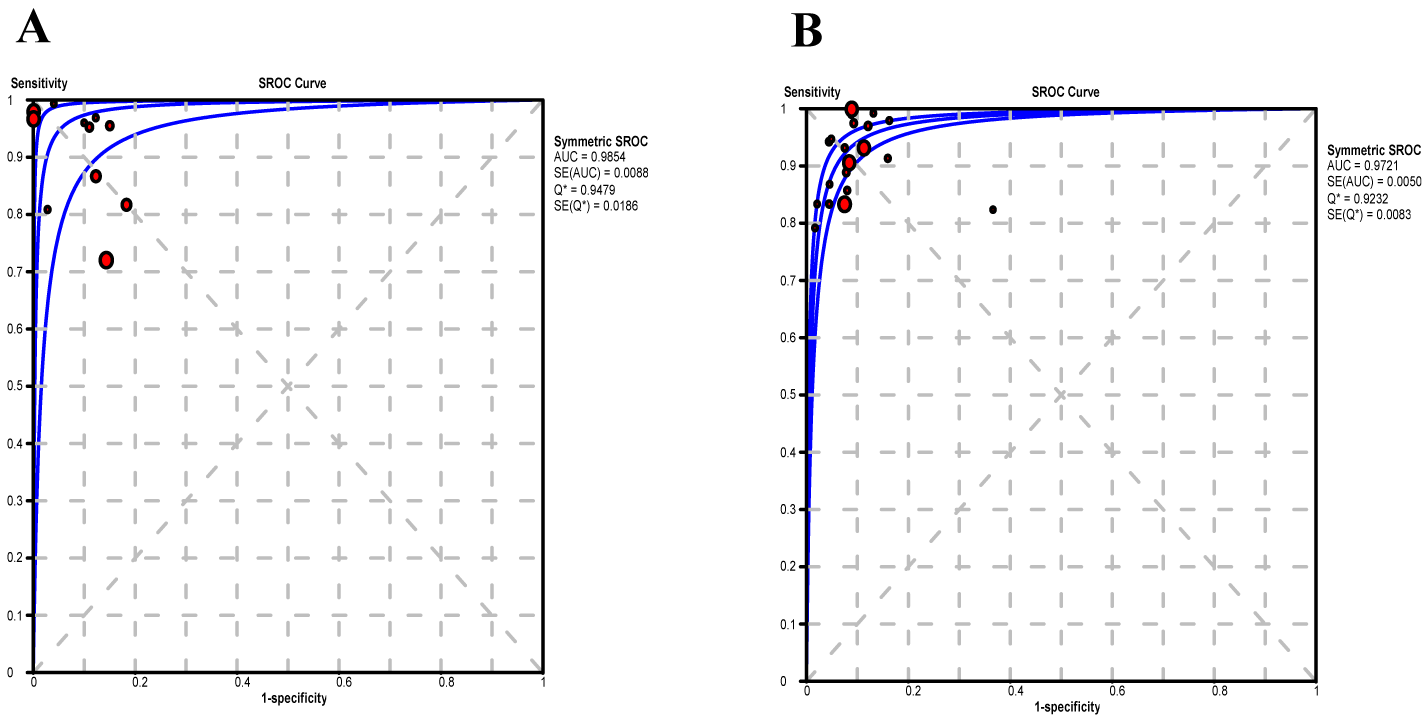


**Supplementary Figure S3**. Summary receiver operating characteristic (SROC) curves for the country subgroup. **(A)** Non-Asian studies. **(B)** Asian studies.


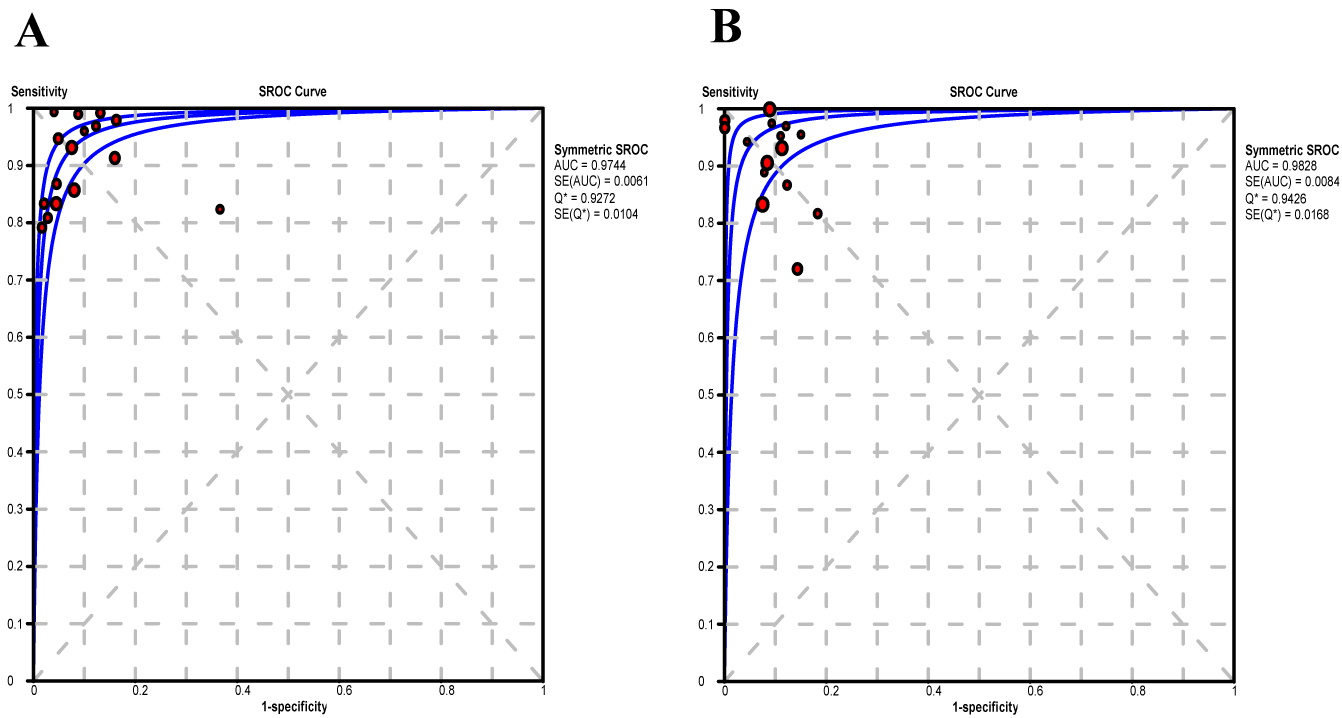


**Supplementary Figure S4**. Summary receiver operating characteristic (SROC) curves for the sample size subgroup. **(A)** Sample size <5000 eyes. **(B)** Sample size >5000 eyes.


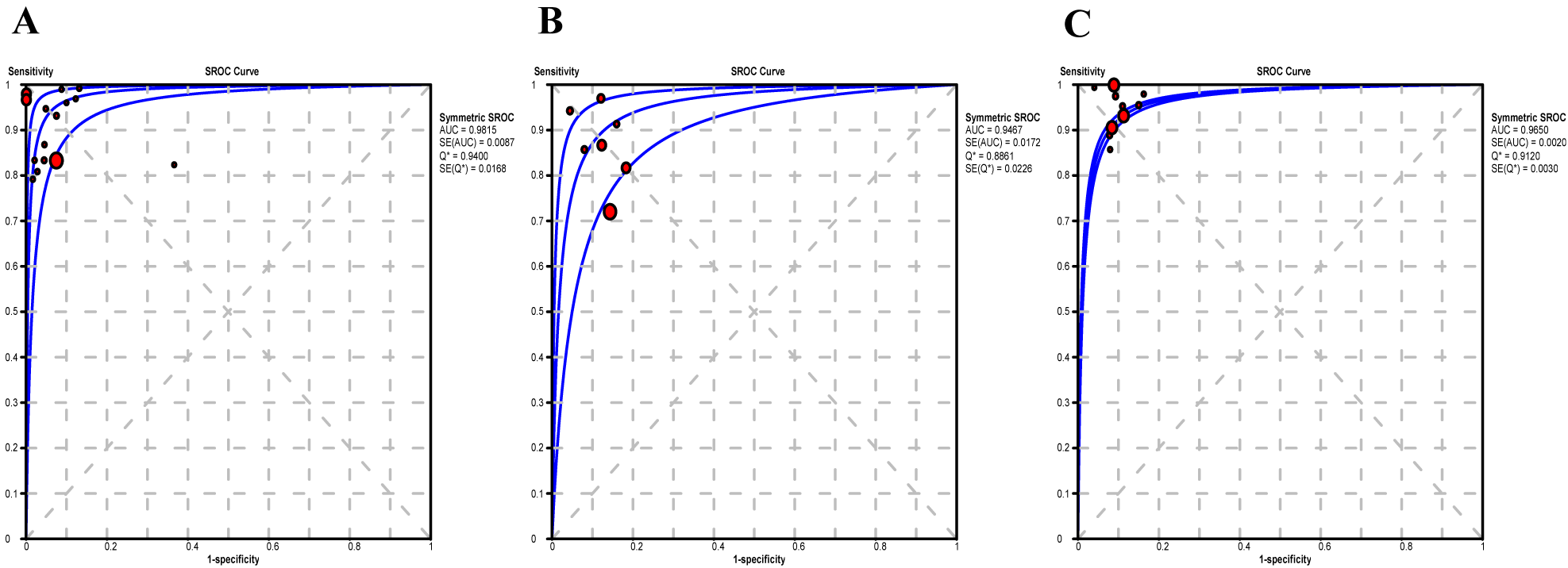


**Supplementary Figure S5**. Summary receiver operating characteristic (SROC) curves for the quality of included studies. **(A)** Low quality. **(B)** Medium quality. **(C)** High quality.


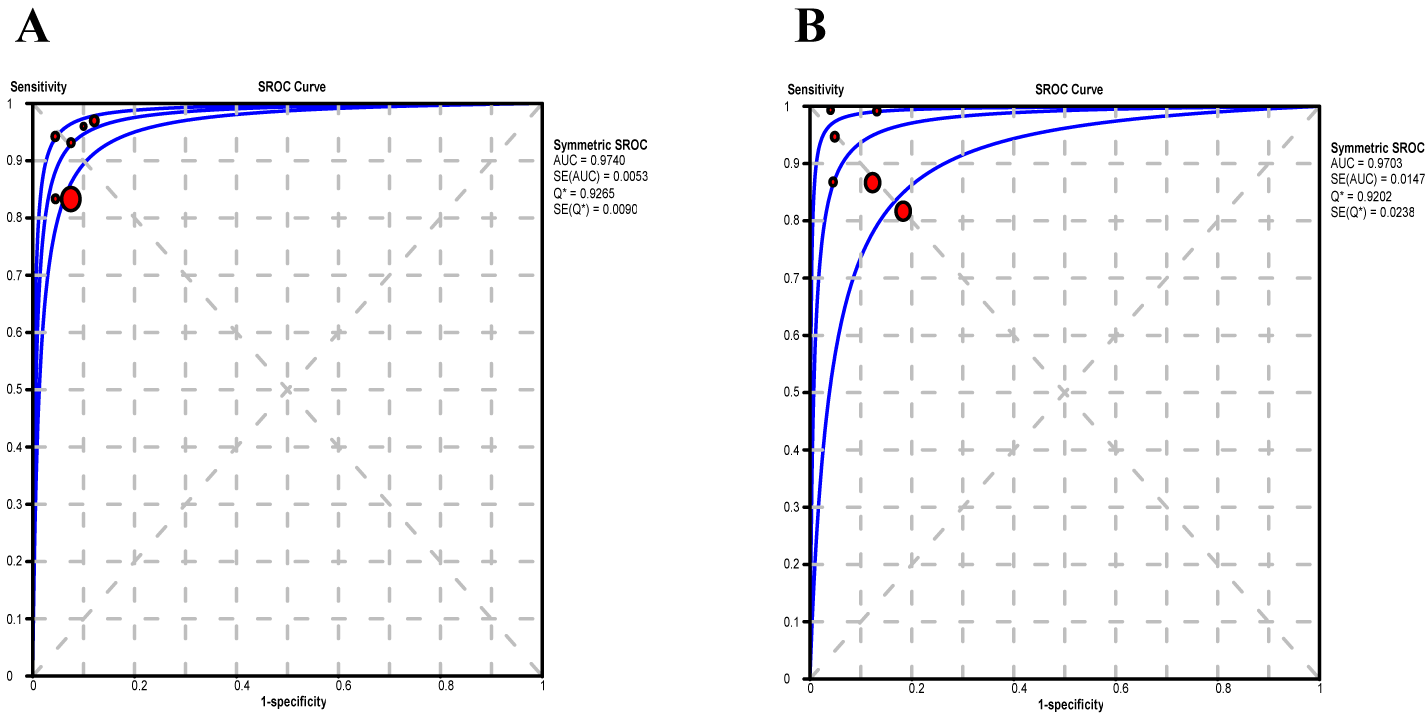


**Supplementary Figure S6**. Summary receiver operating characteristic (SROC) curves for the quality of images. **(A)** <1000
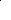
1000 pixels. **(B)** >1000
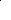
1000 pixels.


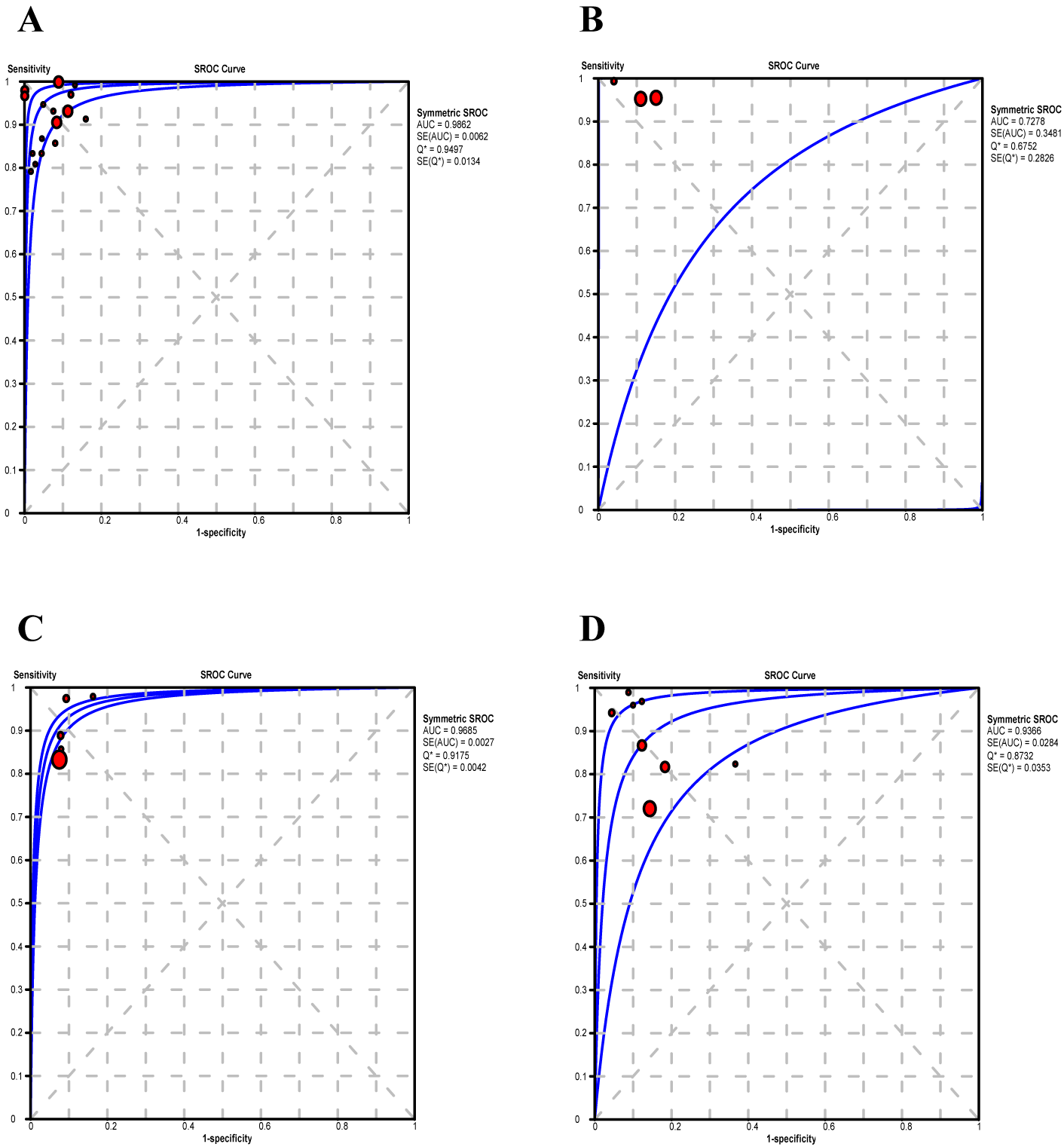


**Supplementary Figure S7**. Summary receiver operating characteristic (SROC) curves for the algorithm. **(A)** Convolutional neural network (CNN) algorithm. **(B)** Machine learning (ML) algorithm. **(C)** Neural network (NN) algorithm. **(D)** Other algorithms.


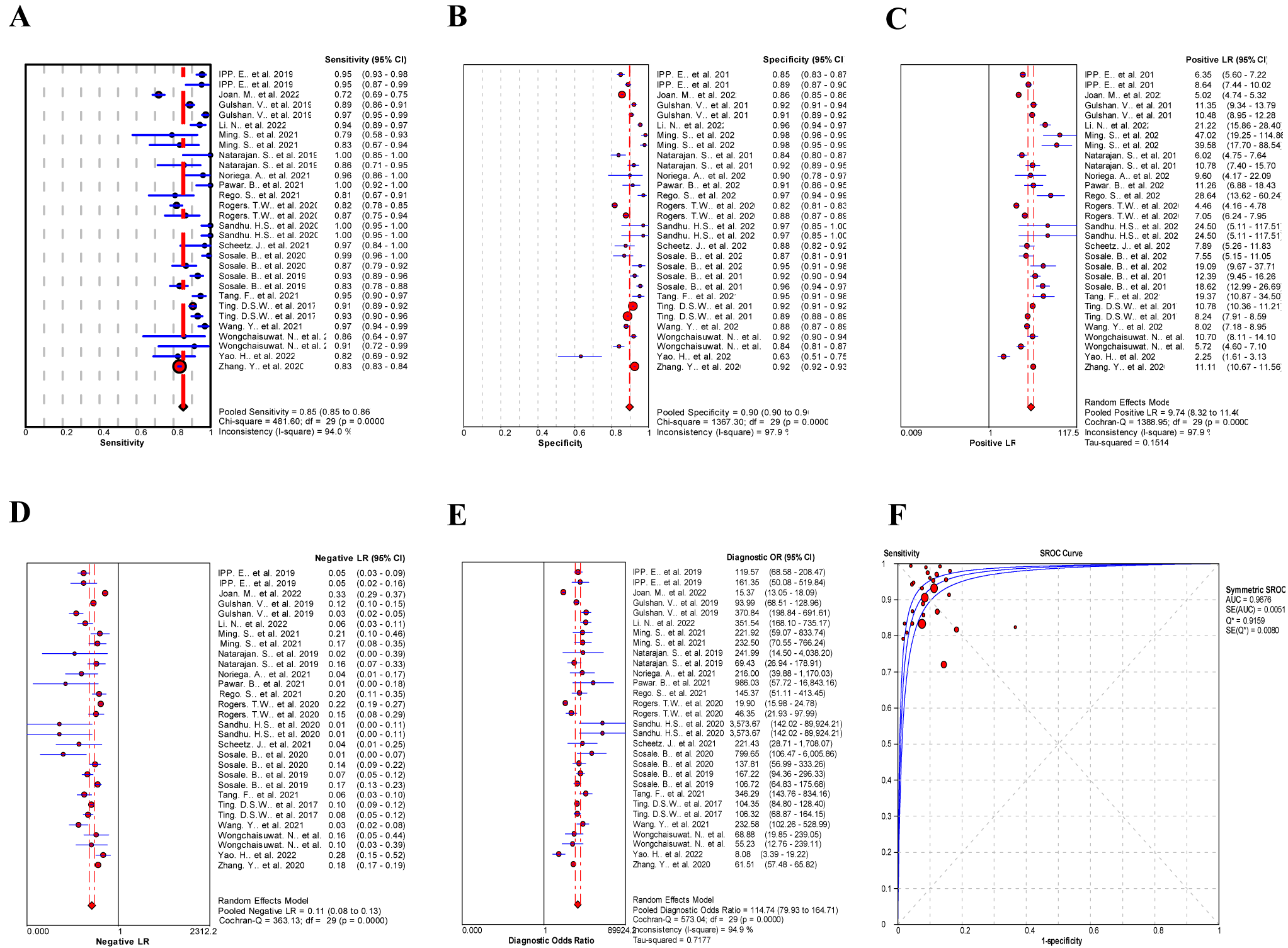


**Supplementary Figure S8**. Meta-analysis results after three sensitive studies were excluded. **(A)** Forest plot of pooled Se. **(B)** Forest plot of pooled Sp. **(C)** Forest plot of pooled positive likelihood ratio (LR+). **(D)** Forest plot of pooled negative likelihood ratio (LR-). **(E)** Forest plot of pooled diagnostic odds ratio (DOR). **(F)** Summary receiver operating characteristic (SROC) plots.


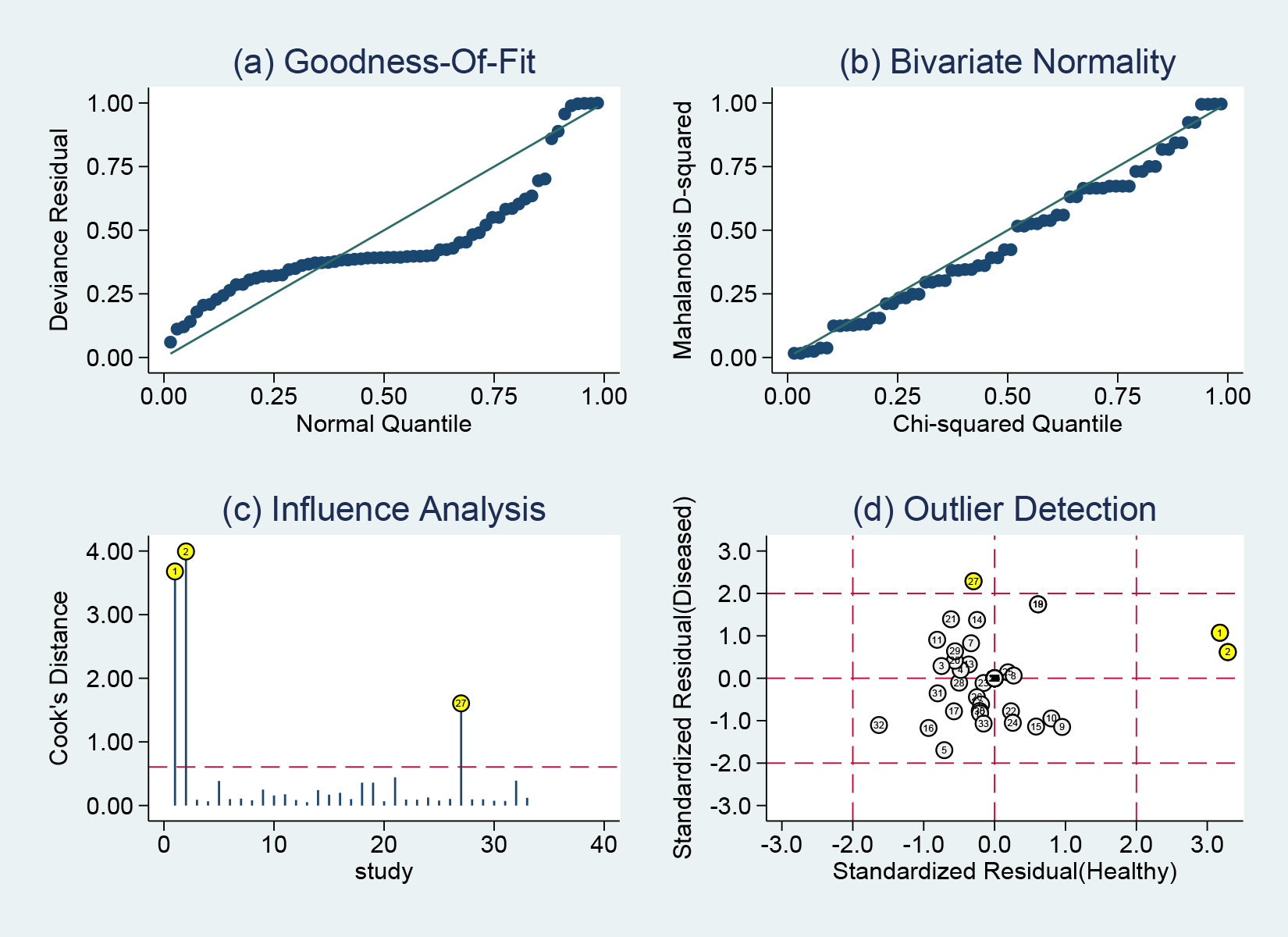


**Supplementary Figure S9**. Results of sensitivity analysis


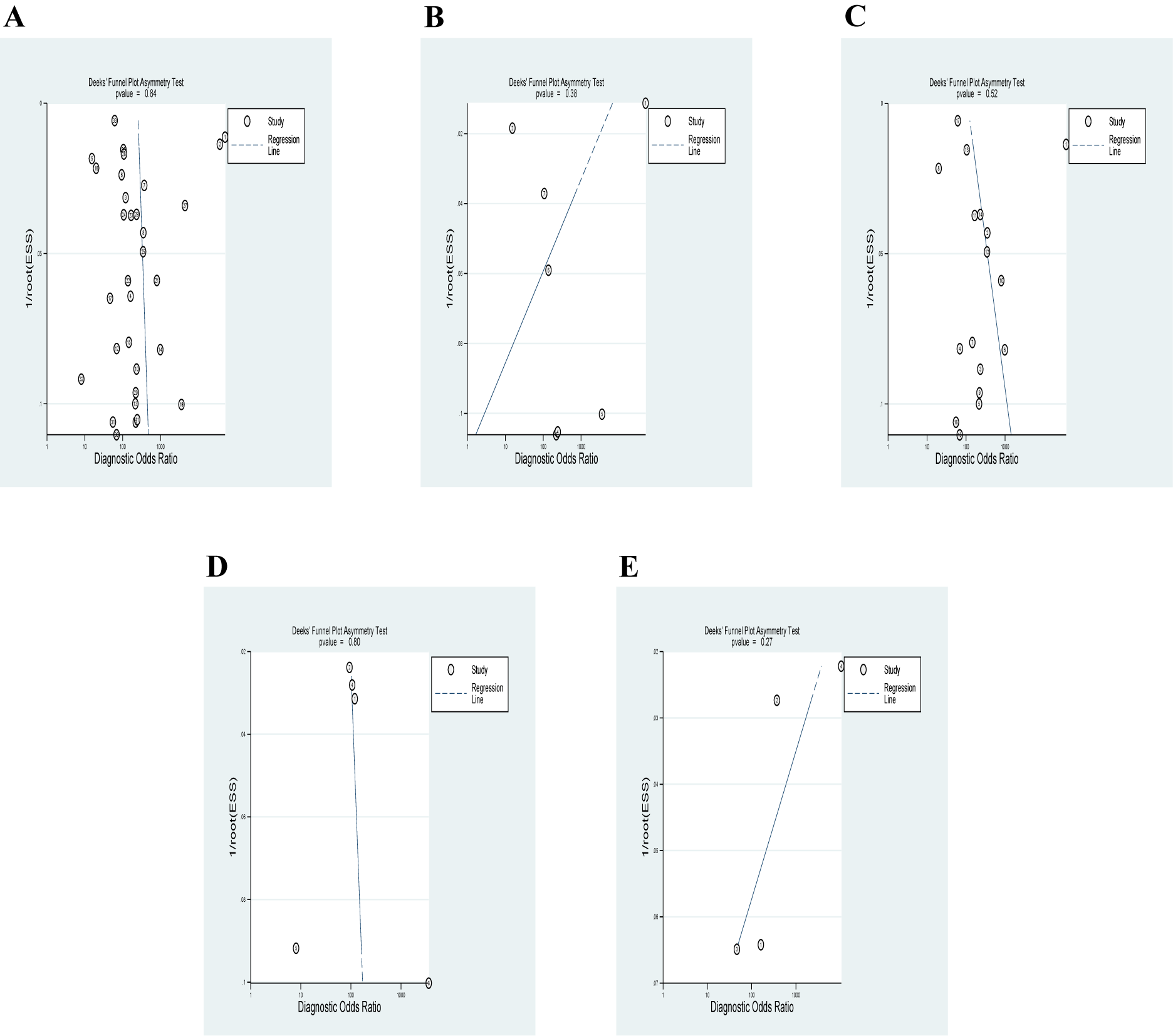


**Supplementary Figure S10**. Deek’s funnel plot. **(A)** All included studies. **(B)** Any diabetic retinopathy (DR). **(C)** Referable DR (RDR). **(D)** More-than-mild DR (mtmDR). **(E)** Vision-threatening DR (VTDR)
